# A Preliminary Exploration of the Impact of Aged Care Reforms on the Governance of Two Australian Residential Care Facilities

**DOI:** 10.1177/23337214231176369

**Published:** 2023-05-23

**Authors:** Cathy Monro, Lynette Mackenzie, Sanetta duToit, Kate O’Loughlin, Lee-Fay Low

**Affiliations:** 1 University of Sydney, NSW, Australia

**Keywords:** Australia, health care reform, nursing homes, quality of care, management

## Abstract

**Objectives:** Australia’s ongoing aged care reforms have re-focused service provisions from a provider-driven policy approach to a consumer-directed care focus and redirected residential care subsidies. This study aimed (i) to identify the experiences and perceptions of people involved in the governance of residential care facilities about their management of changes due to new accreditation requirements and funding mechanisms, and (ii) to describe their strategic responses to aged care reform changes. **Methods:** A qualitative description design used interviews exploring perspectives of Board Chairs, Board Directors, and Chief Executive Officers of two NSW-based residential care organizations. Thematic analysis was conducted of interview transcripts. **Results:** Four key themes emerged from the data: (1) Business strategies and challenges under reform conditions including the need for business diversification and new approaches, (2) costs incurred by the reforms such as compliance with accreditation requirements, (3) workforce demands: for example maintaining staffing levels and training needs, and (4) expectations about maintaining quality of care. **Discussion:** Changes were necessary in business models for facilities to remain sustainable, meet staffing needs, and continue to provide services in a complex, changing fiscal environment. These included generating revenue streams other than government subsidies, better clarity about government support and establishing partnerships.

## What Do We Already Know About This Topic?

Following recent aged care reforms in Australia there is very little known about the impact of these reforms on the governance of nursing homes and how boards and executives have had to adapt to remain financially viable and compliant with new regulations.

## How Does Your Research Contribute to the Field?

This study has confirmed the governance challenges especially for single-site, non-profit providers to deliver high quality and efficient care that is consumer-focused by developing workforce capacity, diversifying income sources, and meeting accreditation requirements.

## What Are Your Research’s Implications Toward Theory, Practice, or Policy?

To adapt to the complex and changing residential care environment changes are necessary in the skills mix of board members responsible for governance, the capacity of the workforce, business models for facilities to remain sustainable, and support for residents and families.

## Introduction

Population aging is a global phenomenon. One in six people internationally will be aged 65 and over by 2050, with the number of people aged 80 and over tripling from 2019 to 2050 (United Nations Department of Economic and Social Affairs, 2019). In Australia, one in four people will be aged 65 or over, with the number of people aged 70 and over more than doubling by 2060 (Productivity Commission, 2013; Treasury, 2021). Australian government expenditure on aged care increased by more than 40% in the last decade, funding approximately 80% of total aged care spending while user contributions made up the balance (Treasury, 2021). Historically, Australia has had a residential aged care-oriented and provider-driven national policy focus and changes from residential to community care dates back to the McLeay Report in the 1980s (Royal Commission into Aged Care Quality and Safety, 2019). In 2012, the government announced the 10-year Living Longer Living Better (LLLB) aged care reforms as a response to population aging and to relieve fiscal pressure (Department of Health, 2012; Productivity Commission, 2013; Treasury, 2015). More recently the Royal Commission into Aged Care Quality and Safety (2021) has expanded age care reforms and governance requirements. These policy changes have impacted the governance of residential aged care facilities and consequently on the availability and quality of care for older people.

The LLLB reforms adopted a Consumer-Directed-Care (CDC) policy approach that shifted the focus from residential aged care provision to meeting the needs of older people for community supported care in the home. This reform approach also aimed to support older people’s desire to remain living in the community for as long as possible (Kendig et al., 2017). While countries including the United States, United Kingdom, Germany, France, and Japan have long adopted the CDC model (Christensen & Pilling, 2019; Lewis & West, 2014; Phillipson et al., 2019; Prgomet et al., 2017), the commencement of LLLB reforms was a new CDC policy approach in Australia. Its intention was to provide control for older people over their care requirements and choice of service providers (Low et al., 2012; Phillipson et al., 2019). Similar to the UK, individual budgets were introduced to enable self-directed support, giving individuals the responsibility of deciding what services and support they needed (Prgomet et al., 2017). Under LLLB reforms, this CDC funding model only applied to homecare services while residential aged care remained under the previous funding model where providers claimed care subsidies from the government through the Aged Care Funding Instrument (ACFI) mechanism (Department of Health, 2017).

However, one of the reform initiatives was to redirect government subsidies from residential care (i.e., ACFI) over 5 years to other aged care reform initiatives such as homecare services and to train the aged care workforce to meet the demand for home and community supported care (Department of Health, 2012). This involved downgrading care subsidy categories and restricting eligibility of ACFI subsidy claims by residential care providers. Furthermore, government regulations also restricted providers in what they could charge the residents for their cost of care (Nusem et al., 2017). Whilst total expenditure on residential aged care consistently increased yearly throughout this period, residential care remained underfunded given the increased demands on services. As a result, providers who operate residential aged care facilities have had to find other sources of income to maintain the same level of quality care for their residents (Nusem et al., 2017).

At the start of LLLB reforms, the federal government provided 71% of total aged care funding to residential aged care providers, of which almost 80% paid for nursing and personal care through ACFI (Aged Care Financing Authority, 2013). Data in July 2021 reported that the total aged care funding allocated to residential aged was reduced to 63% (Aged Care Financing Authority, 2021). Given the ongoing and increasing demand for residential aged care places, residential care providers are faced with the challenge to ensure financial viability while maintaining quality of care for older Australians with increasing care needs. The number of older people entering Australian residential care has increased by 15% over the last 10 years, and older people living in permanent residential aged care are commonly aged 85 to 89, in need of more care and have a shorter length of stay (Australian Institute of Health and Welfare [AIHW], 2021). Most residential aged care facilities in Australia are operated by non-profit organizations (AIHW, 2021). Since the introduction of the CDC model in aged care policy, the number of residential care providers has been in steady decline. Government reports have shown that there were 1,054 residential aged care providers at the start of the LLLB reforms, and by 2019 the number had reduced to 873, suggesting a consolidation of providers (Aged Care Financing Authority, 2013, 2020). Industry reports have highlighted the vulnerable financial position, particularly of smaller, independent residential aged care providers as result of the reforms (StewartBrown, 2020, 2021).

Boards of Directors and Chief Executive Officers (CEOs) are at the governing level of provider organizations’ decision-making processes (Considine et al., 2014). They have the governance responsibility of setting business strategies that ensure financial viability under the reform conditions while maintaining quality care delivery needed and expected by older Australians (Cooper, 2005). Corporate governance is defined as the systems that control an organization and how the organization is accountable. Clinical governance is “an integrated set of leadership behaviours, policies, procedures, responsibilities, relationships, planning, monitoring and improvement mechanisms that are implemented to support safe, quality clinical care and good clinical outcomes for each aged care consumer” (Aged Care Quality and Safety Commission, 2019, p. 3). Despite this important role, studies concerning governance issues have been scarce and there is limited focus on examining the delivery of residential aged care under LLLB reforms from a governance level perspective (Considine et al., 2014; Johnston & Hume, 2015; Nusem et al., 2017). Hough and McGregor-Lowndes (2022) suggest that changes in legislation and regulatory standards in aged care have led to new expectations of boards and directors of service providers. Despite having always had a legal duty to demonstrate care and diligence, and monitoring an organization’s services, the expectation on those responsible for governance (who are often voluntary) has been elevated to include higher expectations of ensuring quality and safety. The recent findings and recommendations made by the Royal Commission into Aged Care Quality and Safety (2021) have highlighted the critical nature of governance and emphasized the need for supporting and enabling providers to ensure governance accountability.

In 2022 a new funding process for residential care was introduced—the Australian National Aged Care Classification (AN-ACC) (Department of Health and Aged Care, 2022). This replaced ACFI and involves a detailed assessment of resident care needs performed by an independent assessor, resulting in the number of care minutes the resident is entitled to. Given the complex governance environment of residential care, and its effect on the decisions that can be made by clinicians and families on the long- term care of older people, this exploratory study aimed (i) to identify the experiences and perceptions of people involved in the governance of residential care facilities during ongoing aged care reforms about their management of changes due to new accreditation requirements and funding mechanisms, and (ii) to describe their strategic responses to aged care reform changes.

## Methods

### Study Design

This study draws on the perspectives of Board Chairs, Board Directors, and CEOs of residential aged care providers to explore the strategic and operational changes required of provider organizations in delivering care under aged care reform conditions. We explored the impact of the reforms on residential care delivery at governance levels for non-profit organizations (representing the majority of aged care facilities in Australia). Study information with invitations to participate were distributed through industry networks and peak bodies such as Aged and Community Services Australia. Retirement villages were excluded from this study as they provide accommodation for older people who do not require the higher level of care offered by residential aged care facilities, and they are not subsidized by the Australian Government (Department of Health and Aged Care, 2020).

The study design utilized qualitative descriptive research methods based on a naturalistic and inductive approach (Bradshaw et al., 2017; Neergaard et al., 2009). A qualitative design was chosen to explore the impact of the current reforms from the perspective of those who were experiencing it without seeking to measure outcomes quantitatively. Semi-structured interviews were used to allow flexibility and spontaneity with participants in describing their understanding and experiences of the reforms (Green & Thorogood, 2014; Neergaard et al., 2009). No repeat interviews were conducted. An intentionally broad interview guide was developed with open-ended questions for exploratory purposes (Liamputtong, 2012). See Table 1 for the interview schedule.

**Table 1.** Interview Schedule.

Can you describe your background and experience in aged care? • Work history and roles • Understanding of the aged care reforms How have the current aged care reforms affected your organization’s governance and strategic directions? • Changes made • Costs • Staffing • Effect on services for residents • Business models • MyAgedCare portal How have you accommodated the accreditation requirements? • Staffing for accreditation preparation • Costs of accreditation • Audits and compliance • Benchmarking The government’s perspective is to provide consumers in residential care with more choice and control. How does this translate into your organization? • Attitudes to spending allocated funds • Minimum services • Extent of choice What feedback have you received, from the staff, clients, and families about the aged care reform changes? • Clarity about the future • Staffing Any other comments you would like to add?

### Data Collection

All interviews were conducted at the participants’ workplace and were conversational and exploratory, allowing the opportunity to gain in-depth information from the participants (Doody & Noonan, 2013). Interviews ranged from 25 to 90 min and were digitally recorded with permission. Those who elected to review the coding were sent a copy for comments (Hagens et al., 2009). There were no further comments from the participants. Field notes addressing any contextual factors during the interviews were kept by the first author who conducted the interviews.

### Data Analysis

All interviews were audio-taped and transcribed verbatim by the first author to allow data immersion, and to ensure that the perspectives of the participants were privileged in the data analysis process (Bradshaw et al., 2017; Saldaña, 2013). The first author kept a field diary following each interview. Using a process of reflective thematic analysis (Braun & Clarke, 2006, 2020), two authors repeatedly read the interview transcripts and coded the dataset independently. Consensus on the codes were reached following discussion and comparisons to ensure coding consistency. A coding book with descriptions was produced to guide the subsequent coding rounds, followed by categorizing themes and subthemes.

### Ethical Considerations

This study was approved by the University of Sydney Human Research Ethics Committees (2017-881).

## Results

Two non-profit organizations responded to the invitation. Two Board Chairs, two Board Directors, and two Chief Executive Officers consented to participate in the study (Table 2). No participants dropped out once recruited.

**Table 2.** Participant Information.

Participant code	Position (period)	Provider location	Business structure
G1	Board Chair (30+ years association with the organization, 2 years as Board Chair)	Central Coast, the State of New South Wales, independent organization at one site	Residential aged care facility, retirement village, with an introduction of homecare services being part of its new business strategy to adapt to the reforms
G2	Board Director (4 years)		
G3	Board Director (long association with the organization from CEO to Board Director)		
G4	CEO (12 months)		
G5	Board Chair (10 years)	Metropolitan Sydney, independent organization at one site	Residential aged care facility, retirement village, with a new Wellness Center providing services to the wider community as part of its business diversification strategy to adapt to the reforms
G6	CEO (8 years)		

Both participating organizations were independent, non-profit, single-site residential aged care providers; one located in Metropolitan Sydney and the other in a regional area in the state of New South Wales. The regional area was introducing homecare services as part of its business strategy to adapt to the reforms’ CDC focus, while the organization in metropolitan Sydney had established a homecare service within their retirement village and created a Wellness Center to increase revenue streams.

Four key themes emerged from the data: (1) Business strategies and challenges under reform conditions, (2) Areas of cost, (3) Staffing issues, and (4) Participants’ perceived expectations. Table 3 summarizes the themes and sub-themes from the data.

**Table 3.** Themes and Sub-themes.

Themes	Sub-themes	Sample quotes
Business strategies and challenges under reform conditions	Business diversification and approaches	“If we were just a residential aged care provider, we wouldn’t survive. . . I’m talking new ventures as well, outside of aged care . . . one is the Wellness Centre that’s predominately aimed at over 55-year-olds . . . and we also have a program called My Companion, which is a community-based program, assisting people to enjoy the community.” (G5 Board Chair). “We are selling a retail product and therefore the business had to look and feel that way.” (G6 CEO). “An aged care facility cannot be a hospital. It’s got to present itself like a destination, a place to live where you can also enjoy the things you’ve enjoyed in life. You need to enjoy them here, have a coffee, have a meal, have a place to share with friends.” (G3 Board Director).
	Challenges for residential care providers	“We don’t know what’s happening with ACFI. We don’t know what’s happening with the accreditation. We don’t know what’s happening with workforce . . . No one knows. I just want clarity going forward . . . It just makes it hard to plan for the future.” (G4 CEO).
Costs incurred by the reforms	The impact of structural reform and initiatives on cost	“We used to get [from the government] “here’s the money, and you spend it”. Now, [the government says] “here’s the potential money, what are the needs of your clients and how are you going to meet them?” It changes your entire focus of your business, to be customer focused.” (G5 Board Chair). “It’s the little things, like no CPI increase in government funding, or 50% indexation and so you know, wages go up by 3% and yet the funding goes up by 1.5%.” (G5 Board Chair)
	Cost to provider mission and values	“If we wanted to make money, we would make it more efficient and get rid of a lot of staff and that’s where the money would come from. But it’s not what we are about.” (G4 CEO).
	Cost of compliance	“You take good people off the floor, and you put them in admin positions to fill out bucket loads of paperwork that really aren’t relevant and don’t have direct impact on the quality of service the client gets . . . The reality is, the staff care for that person in the way they know what that person likes and wants and how they behave . . . they’re not going to refer back to a complex 18-page assessment.” (G6 CEO).
Workforce demands	Changing focus for staff	“The workload is going up. The staff can’t keep up for various reasons. The young ones might not have enough knowledge or skills. The older ones might just not be able to keep up with the physical demand anymore.” (G4 CEO). “People [who] have been [working] in aged care for twenty or thirty years . . . they’re not going to make it unless they change their concept.” (G3 Board Director).
	Staffing challenges	“Staffing levels and retaining staff are hard for us, because there are not many RNs [Registered Nurse] in the area . . . we find it difficult to get good quality RNs, or good quality CSEs [Care Service Employee] because we’re just in a smaller demographic, smaller area.” (G4 CEO). “At the moment you can come and you can work in aged care if you do a six-week course, Certificate III course in aged care . . . That’s just wrong. We need professional standards and the staff have to be able to perform to those standards.” (G3 Board Director).
Expectations about maintaining quality of care	Effective reform systems	“The reforms would be a great new way [to enable providers] to offer something special.” (G6 CEO)
	Maintaining quality care	“There’s a mismatch between what we get funded for, and what the families’ expectations are, huge mismatch, and the government needs to understand that.” (G6 CEO) “They [consumers and families] expect one to one care . . . I’ve had families that write to us or to the Commonwealth Investigation Complaint Scheme because we haven’t put the doilies on a certain way. They expect us to build furniture and put together chairs, have families come and eat with them without charging. They expect a lot. They expect free physio. They expect bus trips every week.” (G6 CEO)

### Theme 1: Business Strategies and Challenges Under Reform Conditions

#### Business Diversification and Approaches

Participants identified changes that were required to their organization’s governance and strategic direction. These involved generating revenue streams other than government subsidies including maximizing opportunities for homecare services, venturing into business outside of aged care services, and establishing partnerships to extend the scope of business.

#### Challenges for Residential Care Providers

Whilst attitudes toward the reforms was generally positive, participants described a lack of clarity over reform initiatives resulting in a level of uncertainty for providers. Some felt that their organizational mission and personal values were now being challenged. They considered their traditional approach to care to be a worthwhile contribution to society and were unwilling to compromise services in caring for older people. For instance, they preferred in-house services over outsourcing to a third party so that they could maintain standards, believing that “*the quality of service (is) down to us, not a third party*” (G5 Board Chair).

There were concerns over the potential increase in premature admission into residential care facilities as result of a shortage in homecare services, and that if the number of providers was reduced because of financial vulnerability, access to residential care in the future would be challenged. With the perceived threat to small independent providers, participants felt that the local community, particularly in regional areas, would be disadvantaged as a result.

Given the structural changes needed for business approaches and diversification, participants commented on the importance of board governance, and of seeking expertise to meet the requirements of a more market driven approach.

### Theme 2: Costs Incurred by the Reforms

#### The Impact of Structural Reform and Initiatives on Cost

Participants acknowledged that the increasing cost of residential aged care due to an aging population would put the government under pressure. However, they highlighted the implications of changing funding focus from providers to consumers. In particular, the reduction in ACFI care subsidies had resulted in consistent operational loss for their organizations. More importantly, participants felt that the implications of a funding focus shift were not acknowledged, and providers were left without sufficient support in managing reform-induced challenges. In addition, implementing the reforms had been time consuming and labor intensive and had to be achieved with less resources.

#### Cost to Provider Mission and Values

Participants also perceived that adapting to reforms impacted the organization’s mission and personal values in their approach to care provision by making them more focused on minimizing costs. One participant highlighted that reducing staff was the only way to minimize costs, which also resulted in outsourcing previous internal services such as catering and laundry (G4 CEO). Some emphasized a personal commitment to maintaining services and support for residents and cautioned against the market driven objectives implied in the reforms (G1 Board Chair, G2 Board Director, G3 Board Director). They felt pressured into refocusing their values from “*mission, vision*” to “*purpose, principles*” to reflect the more “*commercial*” nature of service provision (G6 CEO).

#### Cost of Compliance

Concerns were expressed over increasing costs of compliance since the reforms. These included technological development, staff education and training, monitoring, as well as administrative workload including rewriting policies and procedures to reflect the reforms’ consumer-oriented policy approach (G6 CEO). While technology could be seen as a necessary cost, there were concerns around the complexity of technological development as part of compliance (G5 Board Chair). Increasing regulation such as the accreditation process was considered a high expense (G6 CEO). Others felt that monitoring compliance was less of a priority as the provision of quality care was already part of their organizational structure and practices. Other legislated financial restrictions such as the 28-day cooling off period for residents to make their payments were regarded as a threat to the viability of the organizations (G4 CEO, G6 CEO).

### Theme 3: Workforce Demands

#### Changing Focus for Staff

Participants noted that a significant impact of the reforms was the need for staff to change focus from a relational approach to more performance-driven practices such as the time taken to complete a task and the number of residents they need to assist. One commented that the feedback from staff was that “*they just want to focus on care. They don’t want to know or think that they’re selling a product*” (G6 CEO). Another highlighted the close connection between staff and residents, stating that “*residents regard some of the staff members as friends*,” and that residents would get stressed when those staff leave or are unavailable (G1 Board Chair). There was an increasing level of demand on staff given many residents had higher care needs or were in palliative care where families of residents also needed support (G2 Board Director).

Long-serving staff seemed particularly challenged by the current demands for delivering care. Some staff were resistant to change, resulting from previously entrenched understanding and practice, and a lack of knowledge about related implications (G2 Board Director, G5 Board Chair). Moreover, due to the aging of the existing staff, particularly those that were approaching “*the end of their nursing career*,” there was a reluctance to undertake further training to meet the increasing care needs (G3 Board Director, G4 CEO).

#### Staffing Challenges

Participants emphasized the challenge of staff turnover resulting from business diversification measures such as the departure of key personnel including Director of Nursing and Deputy Director of Nursing as part of organizational restructure (G2 Board Director). Nevertheless, as new business approaches required “*the right people in the right positions*,” the vacancies could be opportunities to recruit appropriate staff (G5 Board Chair). Although the provider in the regional area found it difficult to recruit skilled staff. The training quality for aged care staff was of concern to participants. Moreover, ethical and moral qualities of staff were considered fundamental to delivering quality care (G3 Board Director).

### Theme 4: Expectations About Maintaining Quality of Care

#### Effective Reform Systems

Participants highlighted Australia’s “*good culture*” in “*caring for our aged*,” and that there was a progressive attitude to change in their experience (G1 Board Chair, G3 Board Director). However, they were disappointed by the ineffectiveness of reform initiatives such as the My Aged Care online information portal, and the perceived unrealistic government’s expectation that all older people would be “*tech savvy*” and able to understand and operate new systems (G4 CEO). Clients and families found the portal difficult to understand and operate and required assistance from staff. More administrative staff had to be appointed and the tool was described as “*dysfunctional*” and “*very difficult to navigate*” (G6 CEO).

#### Maintaining Quality Care

Participants generally felt that the government, clients and families expected the provider organizations to maintain a high level of care provision in residential facilities despite ACFI subsidies reduction (G5 Board Chair). In particular, the daily care fee subsidy was viewed as insufficient to cover the broader cost of caring for an older person such as the cost of amenities including electricity and building maintenance (G4 CEO, G6 CEO). Participants believed that families of residents were reluctant to pay for services that no longer qualified for ACFI care subsidies while expecting providers to maintain these services regardless (G3 Board Director, G5 Board Chair). Participants felt their needs as service providers delivering the care had not been met by the government in considering the implications of the reforms (G3 Board Director, G6 CEO).

## Discussion

This study has explored the impact of aged care reforms on the governance and management of two residential care facilities in shifting the policy focus from provider-driven to a CDC approach. We explored a perspective that is currently under-represented in the research literature (Hough & McGregor-Lowndes, 2022). Changes in the funding of residential care services has resulted in a changing emphasis from service provision to financial sustainability for residential care providers. However, there is a disconnect between government policy and consumer expectations of high-quality service delivery under these reform conditions. The capacity of providers to meet the expected care demands is restricted given their financial vulnerability with rising costs. The narratives presented here provide policymakers, clinicians, and families with information about the issues that currently face the governance of residential care facilities.

The marketization of care implicit in the CDC model requires aged care service providers to take a more commercial approach to care by reframing their services as a marketable product, rather than one based on connection and compassion (Nusem et al., 2017). Both provider organizations in the study were challenged to shift from focusing on providing care to shrewd financial management. It presented a conflict between the organizations’ desire for compassion-based care and their financial survival. Davidson (2015) suggested that corporatizing non-profit service providers would lead to an excessive focus on efficiency and profits rather than on the care needs of individuals. As non-profit organizations tend to pursue non-monetary goals and have a level of organizational commitment to social outcomes rather than economic outcomes, the experience of these participants highlights the impact on quality of care of an operational focus on financial viability.

Quality of care is a multidimensional concept involving nursing care as well as interpersonal care or person-centered care, which is related to meeting individual preferences and expectations (Rolland et al., 2011) in the context of an inter-professional team with the older person at the heart of the team (Bhattacharyya et al., 2022). Nursing care such as clinical practice is measurable, while person-centered care such as relationship building between care workers, residents, and families, is not. However, person-centered care contributes at a fundamental level to improving quality of care and accommodates any differences in individual preferences and expectations (Bhattacharyya et al., 2022). As most older people need to be listened to and treated with respect and empathy within residential care settings, a more personalized care planning process is needed that is tailored to individual needs to ensure quality care is provided. This will require a culture shift where residential care staff have time to talk with residents to understand their needs (Coulter & Oldham, 2016).

The strategic focus on measures to mitigate risks of insolvency for providers challenges the sustainability of care quality expected by consumers and required by regulatory guidelines (Nusem et al., 2017). There is a significant monetary cost to achieving both high quality nursing care and interpersonal care. The level of staffing and quality of the staff contribute to quality of care but add to the costs. Those that are in governing positions of residential care providers are faced with the difficult choice between the need to reduce costs and to deliver quality care that is central to their values. For non-profit providers, this conflicting scenario is of great concern (Royal Commission into Aged Care Quality and Safety, 2021). Furthermore, the lack of clarity for residential aged care during reform implementation has impacted the ability of providers to effectively devise appropriate staffing plans to meet the increasing care needs of the residents (Carnell & Paterson, 2017). The ramifications of reduction in subsidies were reflected in the Royal Commission’s (2020, 2021) deliberations in acknowledging that the operations of services were unsupported and underfunded. More specifically, the review of LLLB reforms did not include the impact of ACFI subsidies reduction on service delivery (Department of Health, 2017).

Given the mismatch between government funding structure for residential aged care and the current shortage of aged care workers with the level of skills and knowledge needed to meet the increasing complexity of needs, providers feel constrained in their capacity to attract, (re)train, and retain care workers. This was of particular concern for providers outside of metropolitan areas where there is a general shortage of trained staff. These staffing issues are an ongoing concern and have been raised in reviews of Australia’s aged care system (Aged Care Workforce Strategy Taskforce, 2018; Department of Health, 2017; Monro et al., 2021; Royal Commission into Aged Care Quality and Safety, 2021).

Finally, the COVID-19 pandemic (which occurred after the study data had been collected) brought many of these issues into sharp relief for the governance of residential care facilities. Expanded funding was needed to absorb the costs of personal protective equipment (PPE) and COVID testing for staff, residents and families and costs of providing staff cover for staff unable to work due to illness. Administration costs also increased. The workforce needed to be better trained and many care staff were only permitted to work in one aged care facility. There were also challenges for staff in maintaining quality of person-centered care for older residents who had visiting restrictions imposed on them during lockdowns.

## Limitations

This study explored the governance issues for two non-profit residential aged care providers, that were both single site providers. The study did not involve not-for-profit providers with multiple sites or for-profit providers. Other organizations may have different perspectives and experiences of adapting to the reforms. The sample size was small and could not be generalized to all residential care providers, however a small sample allowed for a deep understanding of the experiences of key informants. The participants had shared experiences across a mix of participant roles (board members, board chairs, and chief executive officers). This was a small population from which to select participants across the two organizations and most eligible people participated. Repeated references made to similar issues suggests that data saturation was reached despite a relatively small group of six participants (Guest et al., 2006; Polit & Beck, 2004). Finally, the study collected data just prior to the COVID-19 outbreak which would have tested the governance of residential care providers further. Given the limited research focus on governance perspective of aged care delivery, this study provides a unique insight and contributes to building an in-depth understanding of the governance aspect of delivering residential aged care.

## Conclusion

This study has provided an understanding of the governance responsibilities and related challenges of delivering care in Australian residential care facilities where there is a mismatch between government funding and consumer expectations of service delivery. The fundamental changes in policy from provider-focused to consumer-driven approaches has resulted in uncertainty and instability for residential aged care providers and challenged their financial viability. This is particularly difficult for small, single-site, non-profit providers. Demand for residential aged care continues to increase, and comprehensive policy considerations are needed to meet the costs of delivering high quality care services that reflect a consumer-directed approach, and to facilitate organizational governance accountability (Royal Commission into Aged Care Quality and Safety, 2021). More research into the effects of reforms on service providers’ organizational governance is needed to understand how efficient and effective aged care service provisions can be ensured.
